# Radiomics Applications in Spleen Imaging: A Systematic Review and Methodological Quality Assessment

**DOI:** 10.3390/diagnostics13162623

**Published:** 2023-08-08

**Authors:** Salvatore Claudio Fanni, Maria Febi, Roberto Francischello, Francesca Pia Caputo, Ilaria Ambrosini, Giacomo Sica, Lorenzo Faggioni, Salvatore Masala, Michele Tonerini, Mariano Scaglione, Dania Cioni, Emanuele Neri

**Affiliations:** 1 Department of Translational Research, Academic Radiology, University of Pisa, 56126 Pisa, Italy; 2 Radiology Unit, Monaldi Hospital, 80131 Napoli, Italy; 3 Department of Medicine, Surgery and Pharmacy, University of Sassari, 07100 Sassari, Italy; 4 Department of Surgical, Medical, Molecular and Critical Area Pathology, University of Pisa, 56124 Pisa, Italy

**Keywords:** radiomics, spleen, machine learning, lymphoma, cirrhosis, gastric cancer, computed tomography

## Abstract

The spleen, often referred to as the “forgotten organ”, plays numerous important roles in various diseases. Recently, there has been an increased interest in the application of radiomics in different areas of medical imaging. This systematic review aims to assess the current state of the art and evaluate the methodological quality of radiomics applications in spleen imaging. A systematic search was conducted on PubMed, Scopus, and Web of Science. All the studies were analyzed, and several characteristics, such as year of publication, research objectives, and number of patients, were collected. The methodological quality was evaluated using the radiomics quality score (RQS). Fourteen articles were ultimately included in this review. The majority of these articles were published in non-radiological journals (78%), utilized computed tomography (CT) for extracting radiomic features (71%), and involved not only the spleen but also other organs for feature extraction (71%). Overall, the included papers achieved an average RQS total score of 9.71 ± 6.37, corresponding to an RQS percentage of 27.77 ± 16.04. In conclusion, radiomics applications in spleen imaging demonstrate promising results in various clinical scenarios. However, despite all the included papers reporting positive outcomes, there is a lack of consistency in the methodological approaches employed.

## 1. Introduction

The spleen, though considered for many years the “forgotten organ”, is well visualized in imaging of the left hypochondriac region of the abdomen [1]. The spleen has a wide range of functions, as it is the body’s larger filter of blood, produces white blood cells and antibodies, and removes microorganisms and inadequate red blood cells [2]. This diverse range of functions of the spleen predisposes it to involvement in a variety of diseases, including immunological, infectious, hematopoietic, storage disorders, and, ultimately, also oncological diseases [3,4]. These can lead to an overwork of the spleen that increases in size, i.e., splenomegaly [3]. Splenomegaly and hypersplenism could also be consequences of chronic liver diseases, through the development of portal hypertension [5]. Furthermore, the spleen can be affected by primary neoplasms, categorized as nonlymphoid and lymphoid, and can serve as a secondary target for metastases, particularly from melanoma, breast, and lung cancers [6]. Imaging modalities such as ultrasound (US), computed tomography (CT), magnetic resonance imaging (MRI), and positron emission tomography (PET) enable radiologists to visualize the spleen’s anatomy and detect gross abnormalities [1]. However, these modalities fail when it comes to capturing the intricate and subtle variations occurring at the microscopic level, which may have the potential to deeply transform our understanding of spleen physiology and pathology.

**Figure 1:**
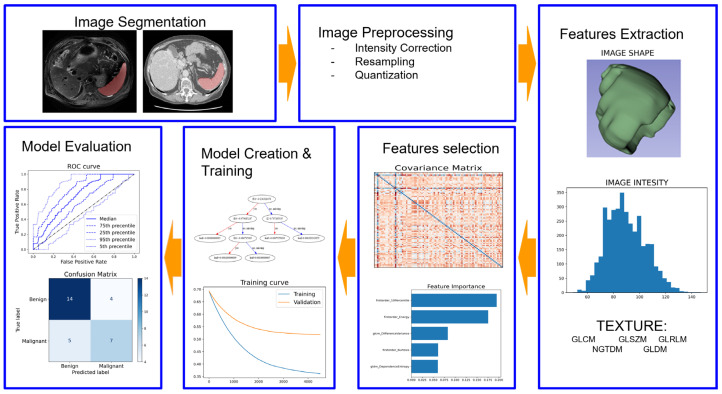
Radiomics pipeline.

Radiomics, defined as the high-throughput extraction of a huge number of quantitative features from medical images, presents an innovative approach to capture these subtle variations and overcome the limitations of traditional imaging [7,8]. The radiomic approach typically involves several key steps, including image segmentation, normalization, feature extraction, model creation, and validation (Figure 1).

Image segmentation is the process of identifying and delineating regions of interest within the image, such as tumors or organs. A wide range of quantitative features is extracted from the segmented regions, which can include shape, texture, and intensity-based features [9]. Finally, these features are used to create predictive models, which need rigorous validation using independent datasets to assess their performance and generalizability, ensuring the reliability of these applications in clinical practice [9]. 

The escalating interest in radiomics research has led to an increase in its applications across various medical imaging domains, particularly in the field of oncology [10,11,12,13,14,15]. Nevertheless, the translation of radiomics within clinical practice is hindered by actual limitations of current research, due to the heterogeneity in all the above-mentioned key steps of the radiomic pipeline, but also the lack of external validation, prospective design, and large-scale multicenter datasets [16,17].

The radiomics quality score (RQS) has been proposed by Lambin et al. to quantify the quality of radiomic research by assessing all the steps of the radiomic pipeline [8].

This systematic review aims to evaluate the state of the art and to assess the methodological quality of radiomics applications in spleen imaging.

## 2. Materials and Methods

### 2.1. Literature Search

To identify all the relevant papers about radiomics applications in spleen imaging, a systematic literature search was independently carried out by two reviewers (S.C.F. and M.F.), accordingly to the Preferred Reporting Items for Systematic Reviews and Meta-analyses (PRISMA) guidelines [18]. The examined electronic archives were PubMed, Scopus, and Web of Science, using as a search string “Radiomics AND Spleen”. The last search was performed on 5 May 2023. Filters were applied to consider only original articles published in English, without any other restrictions. The results were then exported to Rayyan, a cloud-based platform for screening citation data [19]. Duplicates were removed automatically, and all the articles were initially screened by reviewing the title and the abstract. The full texts of the articles that passed this screening were retrieved and read and any disagreement was overcome by discussion to reach a mutual agreement. 

### 2.2. Study Evaluation

All the included articles were examined to extract the following data: year of publication, journal topic (radiological or other), number of patients, disease (primarily splenic or not) imaging technique, targeted organs (spleen or spleen and other organs), feature type (first-order or more), and study design. 

A methodological quality assessment of the included articles was carried out using the RQS tool [8] by two researchers (S.C.F. and M.F.), and any conflict was resolved in consensus to reach a mutual agreement. The RQS tool is made up of 16 items with different scoring ranges in relation to the items’ importance. These items cover the whole radiomic pipeline and are divided into 6 different domains addressing (a) the protocol quality and reporting of imaging at multiple time points and multiple segmentations, (b) the presence/absence of feature reduction and the type of validation, (c) the presence of biological validation and the potential clinical utility, (d) statistical analyses, (e) study design and cost-effectiveness analyses, and (f) evidence of open science and data. 

## 3. Results

After the automated deletion of duplicates (68) and the exclusion of non-inherent reports (15), 15 articles were finally included. 

**Figure 2:**
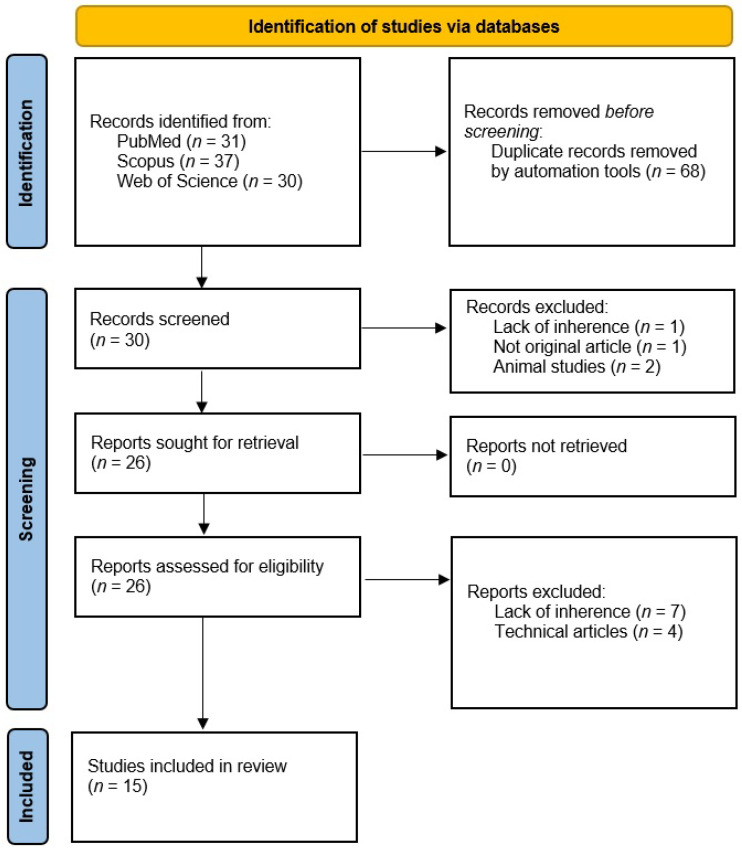
Study selection process flowchart according to the PRISMA Statement 2020 [18].

The flowchart of the systematic search leading to the selection of the articles is shown in Figure 2.

The majority of the articles were published in non-radiological journals (12/15, 80%) starting in 2020. In particular, two articles were published in 2020 (13%), seven in 2021 (46%), four in 2022 (26%), and two in 2023 (13%). In slightly more than half the studies, radiomic features of the spleen were extracted from CT images (11/15, 73%), and the remainder from MR images (3/15, 20%) and from 18F-FDG PET/CT (1/15, 6%). Only two papers addressed a disease primarily involving the spleen (13%), and in 11 studies (73%), radiomic features were extracted also from other organs, particularly the liver and the esophagus. The mean patient number was 231.66 ± 173.60 (range 70–326), and all the studies featured a retrospective design. The characteristics of the included articles are given in Table 1.

Overall, the included papers achieved an RQS total mean of 10.06 ± 6.29 (range −4–16), with a corresponding RQS percentage of 28.70 ± 15.87 (range 0.00–44.44). In the first domain, the majority of the studies (10/15, 66%) described the acquisition protocol, while only in a minority (3/15, 20%) were multiple segmentations carried out, and none collected images at additional time points. Regarding the second domain, feature reduction or adjustment for multiple testing was properly adopted in 14 studies (94%). Of fifteen studies, only in one (6%) was an external validation of the results carried out, while five (33%) did not conduct a definite validation without retraining. In the remainder (9/15, 60%), the validation was based on an internal dataset. Moving to the third domain, all the papers discussed the potential biological correlates of their results, and almost half (7/15, 46%) included a decision curve analysis to assess the potential clinical utility of the developed models. In the fourth domain, addressing statistical analysis, almost all the papers (14/15, 93%) reported discrimination statistics, including three also applying resampling methods. Regarding the fifth domain, none of the included articles reported a cost-effectiveness analysis. Finally, none of the studies made code and data publicly available. The detailed RQS assessment is reported in Table 2.

## 4. Discussion

In recent years, there has been a growing interest in developing radiomic models for implementation in clinical practice, and spleen imaging has not been exempted from this trend. Radiomics has the potential to enhance spleen imaging by providing a quantitative and more objective perspective to the diagnostic process of primary splenic disorders. Enke et al. investigated the role of CT-based radiomics in differentiating malignant lymphoma of the spleen from non-lymphomatous lesions and found an area under the curve (AUC) of 0.86 [21]. In addition, the authors developed a classifier able to differentiate the lymphoma subtypes with AUCs ranging from 0.65 to 0.75.

However, what sets the spleen apart is its central role in immune function and regulation, which consequently leads to its involvement in systemic inflammatory diseases and even in the progression of primary tumors in other organs [1,2,3,4,5]. Therefore, the majority of the articles included in this systematic review did not focus on primary splenic disorders but examined the potential role of features extracted from the spleen in the diagnosis and prognosis of diseases affecting other regions or organs.

Yang et al. developed a radiomics score (Rad-score) to predict the 6-month survival of patients with Hemophagocytic Lymphohistiocytosis (HLH), a rare, life-threatening disorder of immune regulation that can potentially lead to end-organ damage and death [35]. The authors extracted liver and spleen features from CT and PET images obtained from 18F-fluorodeoxyglucose (18F-FDG) PET/CT examinations and combined them with clinical parameters. This resulted in a Rad-score that demonstrated the ability to predict 6-month survival in adult HLH patients, with AUCs of 0.927 and 0.869 in the training and validation cohorts, respectively [32].

The potential role of splenic features and characteristics was also investigated by Wang et al. with a different aim, namely the prediction of the survival rate of patients with early gastric cancer [30]. The radiomic model achieved 80% accuracy in calculating the survival rate. Advanced gastric cancer has also been a subject of research; indeed, Lyu et al. [34] combined gastric cancer and spleen features to predict varying differentiation status. Instead, Pan et al. enhanced the predictive capabilities of radiomics by developing a comprehensive model that combined radiomic spleen features with clinical factors, effectively determining the serosal invasion of gastric cancer [27]. Similarly, the integration of features extracted from multiple organs proved advantageous. Li et al. successfully improved the model’s performance in predicting early and late recurrence of hepatocellular carcinoma after resection by integrating splenic and liver features [23].

Another chronic condition where the spleen plays a significant role in the pathophysiology and complications is liver cirrhosis. Cirrhosis is the final endpoint of multiple liver diseases, such as viral infections and alcoholic or non-alcoholic steatohepatitis, and it has become one of the most common causes of death worldwide [36]. Given the significance and healthcare burden associated with this condition, radiomics has been employed to enhance the diagnosis and staging of cirrhosis and liver diffuse diseases [11,37]. However, cirrhosis is characterized by complex crosstalk between the spleen and liver, as the development of portal hypertension induces alterations in the splenic parenchyma, leading to hypersplenism and splenomegaly [5]. Hence, radiomics of the spleen can potentially offer supplementary insights in liver fibrosis staging, cirrhosis detection and severity assessment, diagnosis of portal hypertension, and prediction of patients at high risk for esophagogastric variceal rebleeding.

Yin et al. found out that machine learning models incorporating CT splenic features outperformed models solely considering CT hepatic features when detecting the liver fibrosis stage [33]. A similar approach was followed by Sack et al. [28], who combined MRI liver and spleen radiomic features to detect cirrhosis, and by Nitsch et al. [26], who developed a predictive model of disease severity for cirrhosis compared with the existing MELD (Model for End-Stage Liver Disease) score.

To assess portal hypertension, Tseng et al. proposed a noninvasive predictive model of portal venous pressure based on liver and spleen CT-extracted radiomic features [29]. The same model was able to effectively predict variceal recurrence (AUC 0.86). 

Esophagogastric variceal bleeding is the most fatal of the consequences of portal hypertension in cirrhotic patients, associated with a high mortality rate [38]. Among the articles included in this review, 4 out of 14 focused on utilizing a radiomics-based model for the diagnosis of high-bleeding-risk esophageal varices in cirrhotic patients [22,24,25,31]. To pursue this objective, the article of Yan et al. used the highest number of patients (796) and extracted liver, esophageal, and liver CT radiomic features [31]. Lijuan Li et al. used the same organs and imaging method as Yan et al., but combined all these features and proposed a radiomics algorithm based on light gradient boosting machine (LightGBM). The LightGBM feature selection showed better performance compared to the other feature selection method used in their work (LASSO, Boruta, XGBoost) [22]. Luo et al. combined radiomics (liver and spleen CT features) and clinical features to create a model to predict esophagogastric variceal bleeding risk with AUCs ranging from 0.925 to 0.912 [24]. Similarly, Meng et al. used liver and spleen CT radiomic features to create a radiomics score to predict the risk of esophageal variceal rebleeding and stratify patients according to the risk of rebleeding probability [25]. 

Finally, another clinical scenario where radiomic features extracted from the spleen were helpful in clinical decision making is COVID-19. The value of lung parenchymal quantitative imaging biomarkers for COVID-19 diagnosis and severity assessment has been already widely proven in the literature [39,40,41]. However, COVID-19 is known to also involve other organs [42]. As highlighted by Batur et al., the spleen may be involved as well, with a decrease in the spleen size and a parenchymal microstructure change in a short follow-up time [20]. However, the clinical relevance of these findings has yet to be demonstrated. 

The methodological quality of the included articles was assessed using the RQS [16]. To our knowledge, the RQS is the only score that has been specifically developed to assess the methodological quality of radiomics studies. The 16 items have been precisely designed to cover the whole radiomics pipeline, allowing us to highlight the presence of critical issues in the assessed papers.

No specific thresholds have been established to categorize work as high or low quality based on this score. However, given the maximum score of 36 (100%), the average score of the reviewed papers, which is 10.06 (28.70%), may indicate suboptimal quality. It is worth noting that this result is not novel. Spadarella et al. already reported a similar overall mean of 18.87% in a paper reviewing all the RQS applications in the literature [43]. Though this result may be partly attributed to the severity of this score, there is no doubt that some recurring issues deserve to be mentioned. To name a few, none of the included studies featured a prospective design, which is considered the highest level of evidence for validating the usefulness of radiomics-based models. Furthermore, only one article provided external validation, which means that the generalizability of these models is yet to be proven. In addition, none of the studies included in the review carried out a cost-effectiveness analysis, nor made data and code publicly available. The scientific community should make an effort to overcome the above-mentioned limitations and unlock the full potential of radiomics in spleen imaging. Large-scale multicentric prospective studies with publicly available data and code may represent a solution to move radiomics forward from scientific research to daily clinical practice. 

## 5. Conclusions

In conclusion, this systematic review highlighted the growing body of literature addressing the potential role of radiomics application in spleen imaging, showcasing promising results in various clinical scenarios. However, it is evident that there is a lack of consistency in methodological approaches across the studies examined. While all the included papers demonstrated positive results, the methodological drawbacks hinder the ability to draw definitive conclusions. By establishing consistent methodologies, we can finally unlock the full potential of radiomics in enhancing our understanding and clinical management of spleen-related pathologies.

## Figures and Tables

**Table 1:** Characteristics of the included articles.

First Author	Years	Aim	Imaging Technique	Number of Patients	Disease	Targeted Organs	Journal Topic	Feature Order	Study Design
Batur [20]	2021	To study changes in the spleen size and textural features of patients with COVID-19	CT	91	Not primarily splenic	Spleen	Not Radiological	First and Higher order	Retrospective
Enke [21]	2022	To investigate spleen radiomic features’ role in differentiating lymphoma subtypes and non-lymphoma	CT	326	Primarily splenic	Spleen	Not Radiological	First and Higher order	Retrospective
Li L. [22]	2021	To identify high- and low-risk EV patients using liver, spleen, and esophagus CT radiomic features	CT	188	Not primarily splenic	Spleen, Liver, and Esophagus	Not Radiological	First and Higher order	Retrospective
Li P. [23]	2021	To explore the usefulness of spleen radiomic features in predicting early and late recurrences of HCC after curative resection	CT	237	Not primarily splenic	Spleen and Liver	Not Radiological	First and Higher order	Retrospective
Luo [24]	2023	To develop a nomogram based on clinical variables and radiomics to predict esophagogastric variceal bleeding in cirrhotic patients	CT	211	Not primarily splenic	Spleen and Liver	Not Radiological	First and Higher order	Retrospective
Meng [25]	2021	To develop a Rad-score from liver and spleen CT images in cirrhotic patients to predict esophageal variceal rebleeding	CT	173	Not primarily splenic	Spleen and Liver	Not Radiological	First and Higher order	Retrospective
Nitsch [26]	2021	To predict disease severity for cirrhosis using liver and spleen MRI radiomic features compared to MELD score and clinical decompensation	MRI	90	Not primarily splenic	Spleen and Liver	Not radiological	First and Higher order	Retrospective
Pan [27]	2021	To develop a radiomic nomogram for preoperative identification of serosal invasion of gastric cancer	CT	315	Not primarily splenic	Spleen	Not Radiological	First and Higher order	Retrospective
Sack [28]	2022	To implement MR radiomic features from liver and spleen to detect liver cirrhosis	MRI	167	Not primarily splenic	Spleen and Liver	Radiological	First and Higher order	Retrospective
Tseng [29]	2020	To propose a noninvasive predictive model of portal hypertension values based on CT radiomic features	CT	169	Not primarily splenic	Spleen and Liver	Radiological	First and Higher order	Retrospective
Wang [30]	2020	To predict gastric cancer prognosis using splenic features	CT	243	Not primarily splenic	Spleen	Not Radiological	First and Higher order	Retrospective
Yan [31]	2022	To develop a radiomic model for diagnosing high bleeding risk esophageal varices in patients with cirrhosis	CT	796	Not primarily splenic	Spleen, Liver, and Esophagus	Not Radiological	First and Higher order	Retrospective
Yang [32]	2021	To predict survival of patients with Adult Hemophagocytic Lymphohistiocytosis by using 18F-FDG PET/CT- radiomic features	18F-FDG PET/CT	70	Primarily splenic	Spleen and Liver	Radiological	First and Higher order	Retrospective
Yin [33]	2022	To combine hepatic and splenic CT radiomic features for liver fibrosis staging	CT	252	Not primarily splenic	Spleen and Liver	Not Radiological	First and Higher order	Retrospective
Lyu [34]	2023	To determine whether radiomic spleen features can be used to distinguish advanced gastric cancer with varying states of differentiation	CT	147	Not primarily splenic	Spleen and Stomach	Not Radiological	First and Higher order	Retrospective

**Table 2:** Detailed RQS of the included articles.

First Author	Item 1	Item 2	Item 3	Item 4	Item 5	Item 6	Item 7	Item 8	Item 9	Item 10	Item 11	Item 12	Item 13	Item 14	Item 15	Item 16	RQS (Total)	RQS (%)
Batur [20]	1	1	0	0	3	1	1	0	0	0	0	−5	0	0	0	0	2	5.56
Enke [21]	0	0	0	0	3	0	1	0	2	0	0	−5	2	0	0	0	3	8.33
Li L. [22]	1	0	0	0	3	0	1	1	1	0	0	3	2	0	0	0	12	33.33
Li P. [23]	1	1	0	0	3	1	1	1	1	1	0	2	2	2	0	0	16	44.44
Luo [24]	1	1	0	0	3	1	1	1	1	1	0	2	2	2	0	0	16	44.44
Meng [25]	1	1	0	0	3	1	1	1	1	1	0	2	2	2	0	0	16	44.44
Nitsch [26]	1	0	0	0	3	0	1	1	2	0	0	−5	2	0	0	0	5	13.89
Pan [27]	0	0	0	0	3	1	1	1	1	1	0	2	2	2	0	0	14	38.89
Sack [28]	0	0	0	0	−3	0	1	0	1	0	0	−5	2	0	0	0	−4	0.00
Tseng [29]	0	0	0	0	3	1	1	1	1	0	0	2	2	0	0	0	11	30.56
Wang [30]	0	0	0	0	3	1	1	1	1	0	0	2	2	0	0	0	11	30.56
Yan [31]	1	0	0	0	3	1	1	0	1	1	0	2	2	2	0	0	14	38.89
Yang [32]	1	0	0	0	3	1	1	1	1	1	0	2	2	2	0	0	15	41.67
Yin [33]	1	0	0	0	3	1	1	0	2	0	0	−5	2	0	0	0	5	13.89
Lyu [34]	1	0	0	0	3	1	1	1	1	1	0	2	2	2	0	0	15	41.67

## Data Availability

No new data were created.
